# Pathological Features of New Animal Models for Primary Biliary Cirrhosis

**DOI:** 10.1155/2012/403954

**Published:** 2011-07-06

**Authors:** Koichi Tsuneyama, Yuki Moritoki, Kentaro Kikuchi, Yasuni Nakanuma

**Affiliations:** ^1^Department of Diagnostic Pathology, Graduate School of Medicine and Pharmaceutical Science, University of Toyama, 2630 Sugitani, Toyama 930-0194, Japan; ^2^Department of Infection, Allergy, Clinical Immunology and Laboratory Medicine, Graduate School of Medicine, Akita University, Hondo, Akita 010-8543, Japan; ^3^Fourth Department of Internal Medicine, Mizonokuchi Hospital, Teikyo University School of Medicine, Takatsu-ku, Kawasaki 213-8507, Japan; ^4^Department of Human Pathology, Graduate School of Medicine, Kanazawa University, Kanazawa 920-8640, Japan

## Abstract

Primary biliary cirrhosis (PBC) is an autoimmune liver disease characterized by immune mediated biliary damage and frequent appearance of autoantibodies against mitochondrial enzymes. There is almost no useful animal model that is globally recognized and routinely used, however, several unique animal models manifested the characteristic clinical and pathological features of human PBC within the last 5 years. Herein, we compare the pathological features of previously reported and newly introduced novel animal models of PBC. Knowledge and understanding of the strengths and the limitations of each animal model have led to the development of promising therapies and novel tools to characterize these clinical conditions. Moreover, suitability of the model for the intended purpose should be confirmed by further research and analysis.

## 1. Introduction

Primary biliary cirrhosis (PBC) is an autoimmune disease of the liver that often develops in middle-aged women. Antimitochondrial antibodies (AMAs) appears in the serum of almost all cases of PBC while the occurrence of AMA is rare in other diseases. The major autoantigens recognized by AMA are identified as the E2 subunits of pyruvate dehydrogenase (PDC-E2), branched-chain 2-oxo acid dehydrogenase (BCOADC-E2), and 2-oxo-glutarate dehydrogenase (OGDC-E2) [1–3]. AMA or anti-PDC-E2 antibody is therefore an extremely useful diagnostic marker of PBC. Pathological destruction of interlobular bile ducts in the liver associated with lymphocytes and plasma cells is known as chronic nonsuppurative destructive cholangitis (CNSDC) and is considered the primary lesion of PBC, and eventually the interlobular bile ducts are destroyed, cholestasis occurs, bile ductules proliferate, and fibrosis develops as the disease advances. In some cases, an epithelioid granuloma is developed in the portal tract accompanied by varying degrees of eosinophilic infiltration [4–12]. PBC is considered a prototype of autoimmune diseases of the liver; it is responsible for both humoral (appearance of AMA/anti-PDC-E2 antibody) and cellular immunity (CNSDC, granuloma formation, etc.). An animal model of PBC that reflects both humoral and cellular immunological features is useful in elucidating the underlying pathophysiology of the disease or establishing an effective treatment. In addition, the characteristic pathophysiological findings of the disease such as the presence of related cytokines and chemokines, nature of inflammatory cells, extent of bile duct destruction, and granuloma formation are considered important aspects of the animal model of PBC. Moreover, ease of handling, the frequency with which relevant pathophysiology develops, and flexibility are important elements. The development of an animal model of PBC has been attempted at many research institutes over a number of years; moreover, some animal models that show pathophysiological symptoms similar to those of PBC have been reported [12–22]. Currently, however, there is almost no useful animal model that is globally accepted and routinely used. Within the last 5 years, there have been also reports of several murine models that manifest the characteristic clinical features of human PBC. In this review, we compare the pathological features of previously reported animal models and a newly introduced novel animal model of PBC. 

## 2. Previously Reported PBC Animal Models of PBC (up to 2005) (Table 2)

Attempts have been made to develop an animal model of PBC in many institutions. In 1989, Krams et al. immunized mice of various strains such as AKR/J, C3 H/J, and CBA/HeJ with recombinant polypeptides of dihydrolipoamide acetyltransferase, which is a constituent of the pyruvate dehydrogenase complex (PDC), and verified that an anti-PDC antibody appeared in each mouse strain although antibody titer levels varied among the strains. However, there were no signs of inflammation or bile duct lesions in the portal tract [13]. Krams et al. transferred lymphocytes from the peripheral blood of a PBC patient into severe combined immunodeficient (SCID) mice, which resulted in the appearance of AMA and anti-PDC-E2 antibodies, as well as marked lymphocytic infiltration around the interlobular bile ducts with slight morphological damage to the portal tract. This was a successful animal model from the perspective of humoral and cellular immunity [14]. However, this model was difficult to reproduce and was only moderately flexible; furthermore, it was not suitable for the analysis of pathological lesions. Masanaga et al. in 1998 used PDC to immunize A/J mice that were neonatally thymectomized, and they succeeded in inducing pathognomonic cholangitis/biliary damage. Although the bile duct lesion in this model was similar to that of human PBC, the appearance of AMA was not clearly described, and the removal of the thymus in the neonatal period posed as a technical difficulty [15, 16]. Tsuneyama et al. [17] and Ohba et al. [18] reported the presence of AMA in the serum of MRL/*lpr* mice, a model of autoimmune disease in which vasculitis, glomerular nephritis, arthritis, inflammation of the salivary glands, and interstitial pneumonia develop spontaneously in the same individual. Because inflammatory cell infiltrates and biliary damage in the portal tract similar to that seen in PBC also appeared in the liver of MRL/*lpr* mice, it was assumed that this mouse may serve as a model of PBC. However, the fact that only about 50% mice showed PBC-like features was a serious problem. The most widespread animal model of PBC is considered to be a graft versus host disease (GVHD) model [19–22]. Initiating the development of GVHD by transferring splenic immune cells from a donor mouse into a host mouse with major histocompatibility complex class-II antigens different from those of the donor leads to the appearance of an underlying autoimmune-like mechanism, such as hypergammaglobulinemia and the production of AMA. Furthermore, the initial pathological changes of PBC with similar associated findings appear in the liver. There have been several pathological analyses using the advantages of this animal model of PBC [19–22]. However, this model is now seldom used, due to its complexity and/or low flexibility.

## 3. Novel Animal Models of PBC (Since 2006) (Tables 3 and 4)

Several novel animal models of PBC have been reported since 2006. These can be roughly classified into spontaneous models, which employ genetic modifications seen in animals, and induced models immunized with xenobiotics whose structures are similar to that of PDC-E2.

Each of these new models shows autoantibodies characteristic of PBC, as well as the appearance of hepatic and bile duct lesions. Their profile also resembles that of PBC with respect to infiltrating inflammatory cells and the appearance of serum inflammatory cytokines. Furthermore, it is easy to establish experimental systems with these models, such as immune cell transfer and mating with other transgenic (Tg) and knockout (KO) mice. Therefore, they fulfill many of the requirements for a PBC animal model, as listed in Table 1, and are currently applied in various investigations of PBC worldwide. Although these animal models are currently considered among the most useful models of PBC, their pathophysiology needs further investigation because some models may show complications that are unusual in PBC, such as peritonitis or inflammatory bowel disease. The pathological features of each of these animal models are outlined below. 

## 4. Spontaneous Models

### 4.1. The NOD.c3c4 Mouse

Nonobese diabetic (NOD) mice are a well-known model exhibiting susceptibility to the spontaneous development of autoimmune insulin-dependent diabetes mellitus (IDDM) [41]. Genetic loci associated with susceptibility to IDDM, as well as several insulin-dependent diabetes (Idd) loci and candidate genes, have been defined through the development of congenic mouse strains [42–44]. NOD mice are also prone to the development of other autoimmune syndromes in addition to IDDM [45]. In the NODc3c4 mouse model, the diabetes susceptibility genes on chromosomes 3 and 4 of the NOD mouse are replaced with the diabetes resistance genes of B6 and B10 mice, respectively. Although this helps in controlling the onset of diabetes in this mouse, autoimmune cholangitis and biliary dilatation similar to that seen in Caroli's disease appear. Serologically, AMA appears in 50–60% and antinuclear autoantibodies (ANA) in 80–90% of the animals. Immunohistochemical analysis demonstrated that the affected parts of the biliary epithelium are infiltrated with CD3^+^, CD4^+^, and CD8^+^ T cells. Furthermore, treatment of NOD.c3c4 mice with monoclonal antibody to CD3 protects them from autoimmune biliary disease. NOD.c3c4-scid mice develop the disease after adoptive transfer of splenocytes or CD4^+^ T cells, demonstrating a central role of T cells in pathogenesis of the disease in this model [23, 24]. Recently, aggregated lymphocytes surrounding the bile ducts resembling the aggregations seen in Sjogren's syndrome, were observed in the salivary glands of this mouse [25]. Because Sjogren's syndrome is often seen as a complication in PBC patients, the pattern of inflammation seen in NOD.c3c4 mice has many similarities to those seen in PBC. However, the cyst-like dilatation of the affected bile duct that is characteristic of these mice is not seen in PBC patients at all. When the dilatation becomes marked, the biliary epithelium of NOD.c3c4 mice frequently exfoliates, and the exfoliated cells together with infiltrated histiocytes fill the lumen. If such dilatation becomes significant, neutrophil infiltration may be also observed resulting in a variable clinical picture such as cholangitis. Therefore, further pathological evaluation of this phenomenon is mandatory.

### 4.2. The Dominant Negative TGF-*β* Receptor II Mouse

Dominant negative TGF-*β* receptor II (dnTGF-*β*RII) mice overexpress the dominant negative form of TGF-*β* receptor type II under the control of the CD4 promoter [46]. Deficiency of TGF-*β* signaling results in various pleiotropic immunological abnormalities including colitis and relatively short lifespan [47–49]. dnTGF-*β*RII mice exhibit major serological and histological characteristics of human PBC, suggesting that the TGF-*β* signaling pathway is important in the pathogenesis of PBC. Serologically, AMA appears in 100% of these mice. The corresponding antigens include PDC-E2, BCOADC-E2, and OGDC-E2; these are the main autoantigens recognized by AMAs of PBC. Furthermore, hepatic lesions characteristic of PBC, such as lymphocytic infiltration, interlobular bile duct destruction, and granuloma formation in the portal tract, appear at high frequency. Various infiltrating cells are found in the portal tracts, including B cells, plasmacytoid dendritic cells, NK cells, and macrophages, in addition to CD4^+^ and CD8^+^ T cells. A particular characteristic is the increased ratio of CD8^+^ T cells to CD3^+^ T cells. This mouse strain presents mild inflammatory bowel disease and crypt abscesses similar to those of ulcerative colitis. Increased levels of inflammatory cytokines such as TNF-*α*, IFN-*γ*, IL-12p40, and IL-6 are also detected in the serum of these mice [26].

The dnTGF-*β*RII mouse is a spontaneous PBC model in which pathophysiological variations are minimum among individuals; furthermore, humoral and cellular immune responses appear to be reproducible and at high frequency. It has given rise to many models that are used for pathophysiological analysis. Yang et al. produced a model by transferring various fractions of splenocytes of dnTGF-*β*RII mice into Rag-1^−/−^ mice. Their study revealed that PBC-like hepatic lesions were produced after the transfer of total splenic lymphocytes and that more severe hepatic lesions occurred after splenic CD8^+^ T-cell transfer. On the other hand, PBC-like hepatic lesions did not appear, however the colitis worsened after splenic CD4^+^ T-cell transfer. Currently, the CD8^+^ T-cell transfer model shows maximum similarities to PBC, such as severe inflammatory cell infiltration, bile duct destruction, and granuloma formation in the portal tract [27]. A derived PBC model, produced by crossing dnTGF-*β*RII mice with a variety of genetically modified mice, is also used for pathological analysis. Moritoki et al. crossed dnTGF-*β*RII mice with mu-mutant mice (Ig*μ* ^−/−^) to produce a B-cell-deficient model in order to study the role of B cells in the pathogenesis of PBC [28]. Chuang et al. studied NKT-cell commitment in a model produced by crossing dnTGF-*β*RII mice with CD1d^−/−^ or CD1d^+/−^ mice [29]. To investigate the roles of various cytokines, Yoshida et al. and Zhang et al. produced animal models by crossing dnTGF-*β*RII mice with IL12p40 KO and IFN-*γ* KO mice and by crossing dnTGF-*β*RII mice with IL-6 KO mice, respectively [30, 31]. Each of these derived models makes a considerable contribution to the pathological analysis of PBC. Interestingly, the grade of hepatic lesions in animal models produced by crossing does not vary greatly among individuals; moreover, it is easy to assign scores to different degrees of pathology for the purpose of evaluation. The dnTGF-*β*RII mouse model is now used in various analyses throughout the world, and it may be regarded as the most useful PBC animal model available at present. However, it is not totally satisfactory as a PBC model because the pathophysiological grade is not severe; furthermore, events that occur in the advanced stage of PBC, such as loss of the bile duct, cholestasis, and fibrosis, are rarely seen. Therefore, the pathophysiological model based on this mouse needs to be further developed.

### 4.3. The IL-2 Receptor *α* ^−/−^ Mouse

IL-2 is critical for the development and peripheral expansion of CD4^+^ CD25^+^ Tregs that promote self-tolerance by *in vivo* suppression of T-cell responses [50, 51]. In IL-2 receptor *α* ^−/−^ (IL-2R-*α* ^−/−^) mice, the IL-2 signal, which is important in controlling the fate of mature T cells, is intercepted; these mice develop an inflammatory bowel disease and a lymphoproliferative autoimmune disease. Also, 25–50% mice develop severe hemolytic anemia at 8–20 weeks of age. It was reported that children with a genetic deficiency of IL-2R-*α* developed clinical manifestations similar to those of PBC [52]. Anti-PDC-E2 antibody is present in the serum of all IL-2R-*α* ^−/−^ mice, and ANA is also present in the serum of 80% of these mice. There is profound lymphocytic infiltration in the portal tract and the interlobular bile duct is also damaged. CD8^+^ T cells are predominant among the infiltrating lymphocytes, and CD4^+^ T and B cells are also present in increased numbers. In addition, granulomas are formed, though in small numbers. Increased levels of inflammatory cytokines such as TNF-*α*, IFN-*γ*, IL-12p40, and IL-6 are present in the serum. The extent of inflammatory bowel disease is relatively severe and frequently associated with formation of crypt abscesses [32]. Using a derived model produced by crossing IL-2R-*α* ^−/−^ mice with CD4 KO and CD8 KO mice, Hsu et al. showed that CD8^+^ T cells participate in the pathogenesis of PBC [33]. The hepatic lesions of IL-2R-*α* ^−/−^ mice are similar to those of PBC, though the complication of severe hemolytic anemia or colitis has not been evaluated. Moreover, the reduced lifespan of these mice makes it difficult to use them in various experiments, such as those involving mating; this is considered a limitation of this model.

### 4.4. The Scurfy Mouse

The Scurfy mouse is a mouse with loss of functional regulatory T cells caused by the *forkhead boxp3* (*Foxp3*) gene mutation. AMAs are present in all Scurfy mice at 3-4 weeks of age, and the portal tract shows moderate to marked lymphocytic infiltration and development of a severe interlobular bile duct lesion similar to PBC with high levels of cytokines such as TNF-*α*, IFN-*γ*, IL-6, IL-12, and IL-23 present in the serum and liver. However, Scurfy mice have an extremely short lifespan of about 4 weeks. This is a serious drawback with regard to its use in experiments [34].

### 4.5. *Ae*2_*a*,*b*^−/−^_ Mice

The anion exchanger (Ae)2_a,b_-deficient mouse model was constructed by Salas et al. in Spain, based on a clinical investigation showing that *Ae2* gene expression was reduced in liver biopsy specimens and blood lymphocytes from patients with PBC [35]. *Ae*2_*a*,*b*^−/−^_ mice exhibit enhanced production of IL-12p70 and IFN-*γ*, an expanded CD8^+^ T cell population, and a reduced number of Treg cells. Serum analysis by immunoblotting showed that 9 out of 11 *Ae*2_*a*,*b*^−/−^_ mice had AMAs. A histological study of liver sections from 11 *Ae*2_*a*,*b*^−/−^_ mice revealed mild to severe portal inflammation in 10 animals. Although the mechanism leading to the deficiency of *AE2* in the liver and blood mononuclear cells in human PBC is unclear, observations of *Ae*2_*a*,*b*^−/−^_ mice indicate a relationship between biliary epithelial dysfunction and the pathogenesis of PBC.

## 5. Immunity Induced by Xenobiotics in Mice

Not only genetic factors but also various environmental factors, such as bacterial infection and exposure to xenobiotics, are strongly implicated in the onset and development of PBC. Most importantly, prolonged exposure over an extended period of time to various xenobiotics with a structure similar to that of the inner lipoyl domain of PDC-E2 has attracted attention as a trigger for the development of PBC. It has been revealed that two types of xenobiotics induce a pathophysiology that is very similar to that of PBC.

### 5.1. Guinea Pigs Immunized with 6-Bromohexanoate

6-Bromohexanoate (6-BH) coupled with bovine serum albumin (BSA) has a structure similar to that of the inner lipoyl domain of PDC-E2 [53]. Increased levels of anti-PDC-E2 antibody, anti-BCOADC-E2 antibody, and anti-OGDC-E2 antibody appear in the serum of guinea pigs when they are immunized with BSA-coupled 6-BH. In addition, slight to moderate lymphocytic infiltration, interlobular bile duct irregularity, and granuloma formation in the portal tract are seen in the liver at an advanced age. Many vacuole-like lipid droplets are seen in the granulomas, and there are also aggregations of macrophages that phagocytoze lipids. Since these vacuolar changes are also seen in control animals to a slight extent, they may be related to immune reactions to foreign substances in the oil emulsion. The limitation of this animal model is the difficulty of use in some experiments because the extent of hepatic lesions is slight and lesions are slow in development, not appearing until 18 months after immunization [36].

### 5.2. Mice Immunized with 2-Octynoic Acid

2-Octynoic acid (2-OA) is a xenobiotic widely used as a food additive and as a component of certain cosmetic products. 2-OA coupled to BSA has a structure similar to that of the inner lipoyl domain of PDC-E2 [54]. Wakabayashi et al. immunized C57BL/6 mice with BSA-coupled 2-OA and detected AMA and anti-PDC-E2 antibodies as well as increased serum levels of TNF-*α* and IFN-*γ*. Marked inflammatory cell infiltration and bile duct lesions in the portal tract frequently appeared, which were mainly associated with CD8^+^ T cells [37]. Using the same methods, Wakabayashi et al. also succeeded in producing a PBC-like lesion in another mouse strain (nonobese diabetic (NOD) congenic strain 1101) [38]. This model is innovative because it induces PBC-like pathophysiology by administration of xenobiotics that may be related to the cause of PBC. As the reproducibility of this model is comparatively high and flexibility is also high, various pathophysiological analyses of this model are in progress in different countries' institutions [39, 40]. At present, however, the hepatic and bile duct lesions show many differences from those of PBC, and further evaluation of the model is needed. In the original immunization procedure used by Wakabayashi et al., BSA-coupled 2-OA was introduced into the abdominal cavity using complete Freund's adjuvant (*M. tuberculosis* in adjuvant oil), following which BSA-coupled 2-OA using incomplete Freund's adjuvant (adjuvant oil only) was administered every 2 weeks as a booster immunization. This model always developed peritonitis of various grades as an adverse effect. A preliminary experiment is necessary in order to check the extent to which peritonitis influences the development of pathological changes in the liver, particularly in that portion which is histologically evaluated. Moreover, a granuloma often appears in the portal tract or the hepatic parenchyma; this could be attributed to the complete Freund's adjuvant administered at the time of immunization. However, methods of immunization have improved; as a result, various modifications designed to induce more serious pathophysiology can be tested. Reports of studies using this model are eagerly awaited.

## 6. Conclusion

The pathophysiology of PBC involves both humoral and cell-mediated immunity, and it can be considered the prototype of autoimmune diseases of the liver. The production of a practical animal model of PBC has been a challenge for researchers interested in PBC and autoimmune diseases for many years. Although animal models based on a variety of mechanisms have been reported from many laboratories, the ideal animal model has still not been available. Recently, several PBC animal models based on different mechanisms were reported. While these newer animal models show the characteristic findings of PBC unlike earlier models, they still show different features from those of human PBC. Understanding the strengths and limitations of each animal model is required in order to match the model to the intended purpose; moreover, suitability of the model for the intended purpose should be confirmed by further research and analysis. 

## Figures and Tables

**Table 1:** Requirements for the ideal animal model of PBC.

(i) Specific liver pathology (cellular immunity)
(1) Destruction of interlobular bile duct
(2) T-cell aggregation around the damaged bile ducts
(3) Epithelioid granuloma formation
(4) Fibrosis/cirrhosis

(ii) Specific autoantibodies (humoral immunity)
(1) Antimitochondrial autoantibodies (AMAs)
(2) Anti-PDC-E2 antibodies, anti-BCOADC-E2 antibodies, and anti-OGDC-E2 antibodies
(3) Antinuclear antibodies (ANAs)

(iii) Other immunological characters
(1) Increase in inflammatory cytokines
(2) Decrease in functional regulatory T cells
(3) Increase in natural killer T (NKT) cells

(iv) General versatility
(1) High reproducibility and disease frequency
(2) Simplicity of model production
(3) Long-term maintenance of disease
(4) Long lifespan without severe complicating disorders

**Table 2:** Representative PBC animal models reported up to 2005.

(1) PDC-immunized mice [13]
(2) Neonatally thymectomized mice with PDC immunization [15, 16]
(3) MRL/*lpr* mice [17, 18]
(4) GVHD model [19–22]

**Table 3:** Novel PBC animal models reported since 2006.

*Spontaneous models*
(1) NOD.c3c4 mice [23–25]
(2) Dominant negative TGF-*β* receptor II mice [26–31]
(3) IL-2 receptor *α* ^−/−^ mice [32, 33]
(4) Scurfy mice [34]
(5) *Ae2_a,b_* ^−/−^ mice [35]

*Xenobiotic-immunized induced model*
(1) 6-Bromohexanoate-immunized guinea pigs [36]
(2) 2-Octynoic acid-immunized mice [37–40]

**Table 4:** Comparison of novel PBC animal models.

	Spontaneous models					Xenobiotic-immunized induced model	
	NOD.c3c4 mice	dnTGF-*β*RII mice	IL-2 R*α* ^−/−^ mice	Scurfy mice	*Ae*2_*a*,*b*^−/−^_ mice	6-BH-immunized guinea pigs	2-OA-immunized mice
*Advantages*							
AMA	50–60%	100%	100%	100%	40–80%	100%	100%
Dominant AMA target protein	PDC-E2	PDC-E2	PDC-E2	PDC-E2	PDC-E2	PDC-E2	PDC-E2
Biliary damage	+	++	+−++	+−++	+−+++	+	+−++
Granuloma	+	+	− or +	−	?	++	++
Pro-inflammatory cytokines	+	+	+	+	+	+	+

*Disadvantages*	Biliary dilatation	moderate colitis	Severe colitis Severe hemolytic anemia	Short lifespan	Late onset	Late onset	peritonitis
